# Is Job Involvement Enough for Achieving Job Satisfaction? The Role of Skills Use and Group Identification

**DOI:** 10.3390/ijerph17124193

**Published:** 2020-06-12

**Authors:** Samuel Fernández-Salinero, Ángel García Collantes, Francisco Rodríguez Cifuentes, Gabriela Topa

**Affiliations:** 1 Psychology Department, Universidad Rey Juan Carlos, 28933 Madrid, Spain; francisco.rcifuentes@urjc.es; 2 University UDIMA and Behavior and Law Foundation, 28400 Madrid, Spain; angel.garcia.c@udima.es; 3 Department of Social and Organizational Psychology, National Distance Education University (UNED), 28040 Madrid, Spain; gtopa@psi.uned.es

**Keywords:** job involvement, job satisfaction, skill use, group identification, occupational health

## Abstract

The main objective of this research is to evaluate the influence of job involvement over job satisfaction mediated through the professional skill use and moderated by group identification. The sample of the current research was composed of 420 subjects. The main results showed that job involvement was strongly related to skill use and group identification. Moreover, the interaction of job involvement and group identification is negatively related with skill use. Our results show that there is no statistically significant relationship between job involvement and job satisfaction. Furthermore, the use of skills is strongly related to job satisfaction. Lastly, we found that a strong group identification tends to harm job satisfaction values.

## 1. Introduction

Nowadays, the organizational world is guided by sustainable principles. Sustainability can be defined as the way of policy making and implementation which is oriented to the inclusion of new generations [1]. 

Commonly, sustainable organizations have three fundamental areas: economic, environmental, and social [1]. Social sustainability has gained importance in the last years, even having created its own category: corporate social responsibility. 

Thus, organizations are more concerned about becoming healthier, enhancing workers well-being, and employee satisfaction [2]. In current organizations, it is a duty to know how to attain the highest level of job satisfaction and job involvement among all the workers [3]. Nowadays, amid COVID-19, we are witnessing workplace innovations that can lead to new challenges. Recent research has shown that it is very important to develop a trade-off between economic efficiency and decent work [4]. Thus, we should be aware of building new innovative organizations with sustainable principles.

At this moment, some enterprises are trying to overcome the COVID-19 crisis by implementing innovations such as teleworking. As recent scholars have stated [4], there are negative consequences for enterprises that do not take social and organizational aspects into account when innovating. These harmful consequences are related to both individual and organizational success. Other recent research has stated that in order to achieve organizational success, social aspects must be considered [5]. Further, innovation success has been related to the desire of workers to contribute to group meetings [6]. As classic research has suggested, when a crisis period appears, survivor stress may lead them to be more involved with their works [7]. A very recent research revealed that colleague support did not have any impact on job involvement [8], but communication quality did. Moreover, colleague support had an impact on job satisfaction. In our research, we did not test the influence of support but of group identity as a social factor that may act as a moderator between involvement and satisfaction.

It is worth noting that social aspects are crucial in the innovation process. Hereto, managers should be aware of paying attention to these social aspects for achieving successful innovations. Recent findings suggest that there is a relationship between social aspects and job involvement [9], as well as on job satisfaction [10].

One of the most desirable outcomes of job innovation is to attain job involvement. Classical definitions of job involvement denote the degree of absorption experienced by an employee in his job activity [11]. Several studies have related job involvement with other variables such as self-esteem, self-efficacy, and work identification [3]. Moreover, job satisfaction is one of the most desirable outcomes for a sustainable organization, as numerous papers [12] have related it to job involvement. In addition, other research papers have associated job involvement with job satisfaction [13]. An interesting question is how these relationships are produced. Both job involvement and job satisfaction have shown negative relations with unhealthy outcomes such as burnout [14].

On the other hand, other research has shown a relationship between job involvement, job satisfaction, and job skills [15,16]. Skill use is defined as the degree that a subject can apply her work conditions to her professional knowledge, skills, motives, and traits [17]. The use of work skills may be an important factor for sustainable organizations. Previous research [16] has shown that professional skill significatively affects important variables such as job satisfaction.

Moreover, recent research has brought to light the importance of organizational and group identity over other important organizational outcomes such as satisfaction, conflict management, or interactional justice [18,19]. Specifically, group identification has been identified as an important modulator in the association with other organizational outcomes. 

Considering the relationship between these variables, our research sheds light on how the relationship between job involvement and job satisfaction occurs. Because job satisfaction is a key variable in the development of healthy and sustainable organizations, and every mentioned variable is related with it, understanding its complexity is the root of this paper. Our research starts from the suggestions of previous research. Firstly, some research [20] has revealed that employee involvement and engagement at work play a crucial role in driving important sustainable organizational outcomes [21]. As a previous study has stated, sustainability increasingly depends on collective involvement of all organizational agents [22]. It has been suggested that sustainability outcomes depend directly on employees’ efforts and behaviors [23] Duarte, Gomes, and Moisés [24] found that companies were concerned with the perception of social responsibility related to job satisfaction. Furthermore, job satisfaction has been recognized as an important outcome of social sustainability [25] and it is a necessary area to research. With this in mind, the main objective of this research is to evaluate the influence of job involvement over job satisfaction mediated by professional skill use and moderated by group identification.

### 1.1. Job Involvement

Job involvement is a complex variable that the scientific literature has defined in numerous ways. Some authors have referred to it as an individual difference, i.e., if a subject’s characteristics fit his job environment [26]. It has also been considered as a stable individual attribute that responds to the current organization. In this research, we consider that this indicator may be related to the way an individual uses her skills.

Other authors have stated that job involvement reflects the importance of an employee work on his entire self-image [27]. Following social identity theory, which will be explained below, part of an individual’s self-image derives from belonging to diverse social groups. Social identity theory has nurtured lots of research, demonstrating that a subject may have numerous identities activated at the same time [28].

Mudrack [29] has stated that work involvement is associated with individual growth in an organization. As Subedi [3] states, job involvement refers to the psychological fact that someone participates in their work, profession, and company. Our question goes deeper and wonders how job involvement affects job satisfaction, especially considering group identification. 

Previous research has shown that job involvement positively affects job satisfaction [30]. In previous research, job involvement has been related to self-image and identity, showing direct and significant relations with professional self-image [31]. With this in mind, we will explore the interaction between job involvement and social identity, as well as its effects on job satisfaction. As will be noted below, social identity refers to the part of an identity that derives from membership of certain groups, demonstrating its importance on organizational outcomes [32].

### 1.2. Job Satisfaction

Job satisfaction is an important variable in sustainable organizations and team management [33]. The classical research regarding job satisfaction have defined the concept as a positive affective response towards the job as a whole [34]. Moreover, Spector [35] stated that job satisfaction refers to the level that an employee likes or does not like his job and its facets. There are multiple aspects that may determine job satisfaction. Because of this, complex and interactional models are needed. 

Some researchers have stated that there may be dispositional individual and contextual variables related to job satisfaction. In this research, we think that, based in previous research [36,37,38,39], job satisfaction may be referred to a combination of individual traits and contextual job characteristics. 

Even though job satisfaction may be composed by three components—affective, behavioral, and cognitive [40]—job satisfaction has been addressed most commonly from its affective facet. Some authors have tried to difference two kinds of affective job satisfaction: global job satisfaction and job facet satisfaction [41]. Specifically, job satisfaction has been addressed the literature as global job satisfaction [3].

Global job satisfaction is composed of multiple factors regarding different job facets such as benefits, relationships, role, hiring conditions, and more. What makes job satisfaction important is its relationships with desirable organizational outcomes like mental and physical health [42]. Moreover, there is enough evidence to affirm that job satisfaction is an important variable in enhancing organizational performance [43]. In this research, we have decided to focus on affective job satisfaction. Based on Thompson and Phua’s research, we locate the difference between rational and affective satisfaction [33]. Further, we assume that job conditions and circumstances evoke an emotional mood and positive feelings, while cognitive satisfaction is related to rational and logical thinking. We are interested in evaluating the emotional response of job involvement, wherein a subject uses their skills. Lastly, we are interested in evaluating how the affective response impacts one’s when people identify them with their workgroup. 

### 1.3. Professional Skill Use

Professional skills have become an important variable in human resource (HR) management. Even some HR directors have recognized them as a key factor in the recruiting process [44]. 

This construct includes lots of important factors such as professional knowledge, high-level skills, critical thinking, teamwork, communication skills, and permanent learning [45]. Previous articles have shown that knowledge is one of the most important skills, followed by personal characteristics and communication [16]. Other research has suggested that organizations with a goal of achieving top performance should recognize individual skills in order to incorporate individuals into the performance appraisal [46].

As the work environment evolves, employers have developed competency-based methods to enhance performance. It has been suggested that it is essential to create a work environment where workers use their skills to achieve job satisfaction. Recent papers have confirmed the relation between work skills use and job satisfaction [47]. Recent research in the information technology field have shown that organizational skills lead to job satisfaction [48].

As Romero and Jiménez [49] have stated, skill use is not widely studied in organizational scientific literature. Some research suggests that people with higher skills tend to show lower levels of job satisfaction [50,51]. However, it has been found that lower skill levels are related to lower levels of job satisfaction. Due to these results, this paper considers if skill use may work as a mediator between job involvement and job satisfaction.

### 1.4. Group Identification

Organizations have changed in recent decades and nowadays are composed of numerous groups and departments that lead to different identification processes [19,52]. 

Social identity theory states that part of an individual self-concept is originated because of belonging to certain social groups. Individuals strive to achieve a positive social identity. After the classical postulates of the social identity theory, the self-categorization theory emerged [28]. Within the optic of this theory, it is posed that personality is structured in a hierarchical form. A subject’s cognitive, emotional. and behavioral processes may be affected by which personality (individual or social) is activated. 

In the organizational field, workers may behave based on their individual or social attributes. Recent research has addressed how identifying with a group may elicit different outcomes on job satisfaction [53]. Specifically, managing organizational identity has been demonstrated to be an interesting variable on influencing job satisfaction [54]. It is important to note that social connection and being a part of a social network are not synonymous. Proof of this claim is that having a social contact did not impact job satisfaction in previous research, whereas identifying with fellow workers did. Other research has found similar positive relations between organizational identification and job satisfaction [55]. 

Organizational identification refers to the sense of belonging within the same organization. Despite the importance of this variable, little research has been conducted to examine the mechanisms between identification and organizational outcomes [56]. In previous researches scholars found indirect effects of organizational identification and job satisfaction [56]. Social identification is linked to positive social identity through motivational and behavioral processes. Subjects did not act according to their personal identity and instead perceived their actions based on their social identity [32]. 

Previous research has emphasized that job involvement positively affects supervisor support and is negatively affected by family and friends [14]. This suggests that social interaction acts differently with diverse groups. Within the same organization can exist different social identities, which can lead to different organizational processes and outcomes [50]. Group identification has sometimes impaired desirable organizational outcomes such as job satisfaction and enhancing intergroup conflict [57]. Since social identity indirectly impacts organizational outcomes, one of the objectives of this paper is to answer the following: does impairing moderation exist in the relationship between job involvement and job satisfaction, especially when considering skill application?

### 1.5. Hypotheses

**Figure 1:**
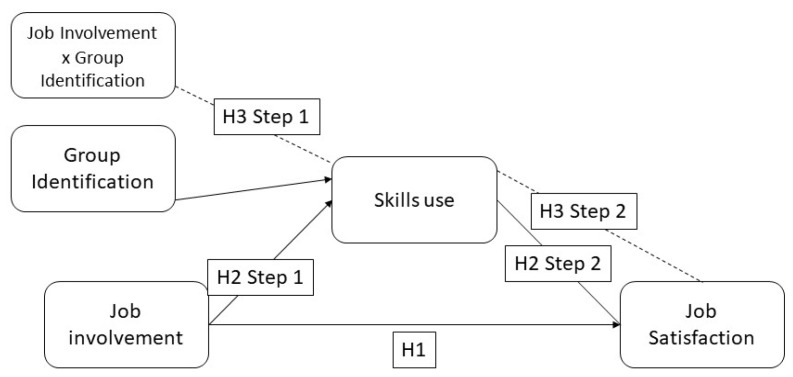
Hypothesis’ graphical model showing the relationships between variables.

The main aim of the current research was to evaluate the impact of workgroup identification in the relationship between work involvement and job satisfaction. Do so, we primarily considered the mediating role of skill use. First, we propose that work involvement has a statistically significant effect on job satisfaction (H1). Moreover, we state that that relationship is be mediated by skill use (H2). Finally, the above-mentioned relations are moderated by group identification (H3). All of these relationships are shown in Figure 1.

## 2. Materials and Methods 

### 2.1. Sample

Several Spanish organizations were invited to complete the questionnaire developed for this research. These organizations came from different parts of Spain and belonged to different organizational contexts (e.g., production, services, transportation, counselling). Data were acquired via the Internet. Anonymity and confidentiality were guaranteed. Participation was voluntary when using the questionnaire’s link and thus we do not have data of the participation rate. The sample of the current research was composed of 420 subjects; 49% were men and 51% were women. The mean age was 40 years old (10 SD). Related to the academic level, our sample was composed by 47.9% of subjects with a university degree. In total, 8.6% of the sample had basic studies, 8.1% had completed high school, 19.3% had vocational training, 15.2% had a master’s degree, and 0.7% had a PhD. With respect to contract type, in our sample, 79.8% had a temporary contract and 21.2% had an indefinite contract. 

### 2.2. Instruments

The questionnaire used in this research was composed of the instruments noted below. 

Sociodemographic: Different sociodemographic variables were included in the questionnaire. For guaranteeing confidentiality, we collected age, gender, academical level, contract type, number of dependents, and seniority at work. 

Job involvement: the job involvement questionnaire [58] was used. In our sample, the reliability measurement value was 0.85. This questionnaire was composed of four items (e.g., “I have very strong ties with my present job which would be very difficult to break”) that saturated one factor. It used a Likert-type scale from 1 (strongly disagree) to 5 (strongly agree).

Skills use: In order to assess this variable, we used the skill discretion subscale from the job content questionnaire (JCQ). As previous research has shown [59], the “decision latitude” subscale refers to the ability of making decisions about one job and the possibility of being creative and using skills being formed by two subscales, i.e., decision authority and skill discretion. Specifically, this last subdimension refers to the flexibility held by workers who decide what skills can be used to best perform their job [45]. Our sample reliability value was 0.76. This questionnaire was composed of six items (e.g., “My job requires me to be creative”) that saturated one factor. It used a Likert-type scale from 1 (strongly disagree) to 5 (strongly agree).

Group identity: In the measurement of this variable, we used Mael and Ashforth’s [60] organizational identification questionnaire. The group identity factor is composed of four items (e.g., “When I talk about this group, I usually say «we» rather than «they»”) that showed a reliability value of 0.88. The instrument used a Likert-type scale from 1 (strongly disagree) to 5 (strongly agree).

Job satisfaction: For the assessment of this variable we used the brief index of affective job satisfaction (BIAJS) [33]. This questionnaire was composed of seven items (e.g., “I like my job better than the average person”) and three of them were distractors (e.g., “My job is time consuming”). In our sample, the reliability value was 0.75, which is acceptable for research purposes. The instrument used a Likert-type scale from 1 (strongly disagree) to 5 (strongly agree).

## 3. Results

**Figure 2:**
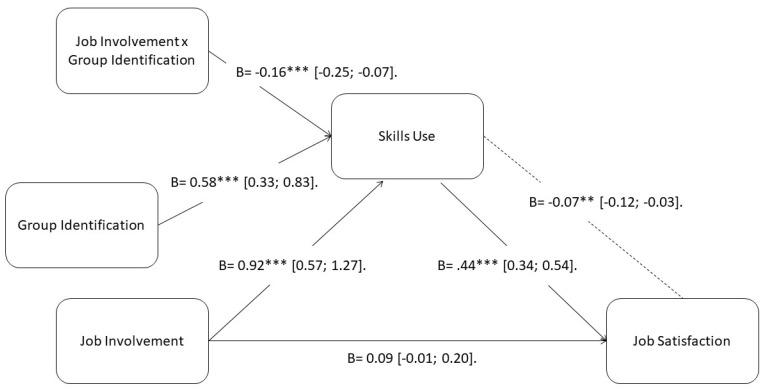
Statistical model where coefficients and effect sizes can be observed. Note: *** *p* < 0.001, ** *p* < 0.01.

In order to assess our hypotheses, we used a moderated mediation model that followed Hayes model 7, developed for the PROCESS macro. The first step for testing the model was to evaluate the correlations between variables. When conducting the data analysis, age, gender, and academical level did not show significant relationships with the rest of the variables. Due to this, they were not included in the final model. The correlation matrix is noted in Table 1. The PROCESS macro used a bootstrapping procedure, which extracted 1000 random samples from the original data. In order to clarify the results of the analyses, Figure 2 shows a graphic display.

### 3.1. Mediation Model

First, we evaluated the direct effect of job involvement over job satisfaction and found that the direct relationship was not statistically significant (B = 0.95, SE = 0.05, 95% CI (−0.01; 0.20), *p* > 0.05). However, skill use was statistically significant when related to job satisfaction (B = 0.44, SE = 0.05, 95% CI [0.34; 0.54], *p* < 0.001). Simple mediation was supported (B = 0.16, SE = 0.03, 95% CI (0.10; 0.22)).

### 3.2. Moderation Analysis

Following the next step of our model, we tested if skill use (M) was moderated by group identification (W) when there was job involvement (X). Our results supported this hypothesis of the interaction negative (B = −0.16, SE= 0.05, 95% CI (−0.25; −0.07), *p* < 0.001). Concretely, at lower values of group identification, skill use was higher. The moderation model was supported at every level of the moderator (*p* < 0.001). At the 16th percentile level, the coefficient was B = 0.47, SE = 0.06, 95% CI (0.35; 0.60). However, at the 84th percentile level, the coefficient was B = 0.23, SE = 0.05, 95% CI (0.12; 0.34).

### 3.3. Moderated Mediation Analysis

Lastly, when assessing the moderated mediation, we affirmed that it was statistically significant. Job involvement (X) showed an impact over job satisfaction (Y). Moreover, the relationship with skill use (M) was moderated by group identification (W) (B = −0.07, SE= 0.02, 95% CI (−0.12; −0.03)). Specifically, at lower levels of group identification, job satisfaction values were higher. At the 16th percentile level, the coefficient was B = 0.21, SE = 0.04, 95% CI (0.13; 0.29). At the 84th percentile level, the coefficient was B = 0.10, SE = 0.03, 95% CI (0.05; 0.16).

## 4. Discussion and Conclusion

The main objective of this research was to explore the relationship between work involvement and job satisfaction, taking the mediating role of skill use into account and assessing the moderating effect of group identification. Considering socially responsible organizations, we chose job satisfaction as a desirable criterion variable. As Moldavanova [1] stated, social sustainability plays a crucial role on the sustainable organizational field. 

Our results showed that job involvement had a statistically significant relationship to skill use and group identification. This is consistent with previous research that relates job involvement and skill use [61]. One interesting aspect that deserves to be studied in the future is that, in our research, we obtained that the interaction of job involvement and group identification is negatively related to skill use. Looking closely at the results, when a subject identifies with her workgroup, she might feel farther from the organizational as a whole. Future research must shed light on the particularities of this phenomena, as well as on the effects of diverse group performance characteristics.

When considering job satisfaction, our research shows that there is no statistically significant relationship between job involvement and job satisfaction. In our research, Pearson’s correlation showed a statistically significant relationships between these variables, but showed no direct relationship. This may be explained by the fact that job involvement needs a way of expression in the workplace. 

The use of skills is strongly related to job satisfaction, which dramatically raises the values of the criterion variable. As the mediation model was significant, our research offers a new optic for managing sustainable organizations, which is aligned with the literature that explains the relationship between overqualifications that hinder job satisfaction [62]. In our sample, subjects were motivated and involved in their works, but also needed to have the opportunity to use their skills. As previous research has shown, job satisfaction can impact job involvement, creating a circular model. Further research should investigate this cycle to try to determinate causality.

Finally, our research shed light on how poor group identification may be connected to skill use. Strong group identification tends to harm job satisfaction values. This is in line with previous research that associated group identification and negative outcomes with organizational conflict [18,52]. It is interesting that although some subjects may need social identification to use their professional skills, group identification may harm job satisfaction. As we posed in the introduction section, feeling socially connected is not the same as perceiving oneself a valued member of a group. There is evidence that identifying oneself as a member of a workgroup instead of an organization raises conflict [55] and thus a subject may use their competences with their workgroup, but not with the organization as a whole. Future research should focus on evaluating how the model results can include organizational identity instead of group identity.

Our research has some limitations. First, a transversal research was designed and it would be desirable to develop longitudinal researches in order to gain more precision in our statements. On the other hand, the use of self-reported measures may bias the results. Job involvement is a desirable variable that may affect results. 

Future research should unmask underlying processes of group identification. Identity should be handled wisely for achieving positive organizational results. There may be some incongruence between personal and organizational perspectives that may activate dissonance. As we know from the self-categorization theory, multiple identities may be activated at same time and previous results have shown that an organization may have different workgroup characteristics [63].

Managers should be aware of these organizational characteristics in order to handle identity properly, leading consequently to job satisfaction. More research on social identity must be carried out in order to achieve a deeper understanding of these phenomena, which can shed light on the different identities, which can foster a healthier work environment.

## Figures and Tables

**Table 1:** Pearson correlation matrix.

	M	SD	1	2	3	4
Age	40	10	-	-	-	-
Job involvement	2.61	0.76	0.01	-	-	-
Job satisfaction	3.39	0.85	0.05	0.23 **	-	-
Skills use	3.49	0.78	0.09	0.35 **	0.43 **	-
Group identification	3.52	0.87	0.07	0.14 **	0.45 **	0.24 **

Note: ** *p* < 0.01. M: Mean; SD = Standard Deviation.
